# 2025 SMFM Oral Schedule

**DOI:** 10.1002/pmf2.12038

**Published:** 2025-01-05

**Table:** 

Thursday, January 30, 2025 • 8:00 AM – 10:00 AM		
Time	Abstract	Oral Plenary Session 1
8:00 AM	1	**Antenatal corticosteroid in twin pregnancy at risk of late preterm birth: A randomized controlled trial** **Seung Mi Lee^1^**; Hyun Soo Park^2^; Soo Ran Choi^3^; Jeesun Lee^1^; Hyun Ji Kim^4^; Jee Yoon Park^4^; Kyung Joon Oh^4^; Geum Joon Cho^5^; Min‐Jeong Oh^5^; Jin Hoon Chung^6^; Sun Min Kim^7^; Byoung Jae Kim^8^; Suk Young Kim^9^; Subeen Hong^10^; Young Mi Jung^5^; Se Jin Lee^11^; Ji Su Seong^12^; Haemin Kim^13^; Sohee Oh^8^; Joongyub Lee^1^; Ji Hoi Kim^1^; Hee Young Cho^14^; Chan‐Wook Park^1^; Joong Shin Park^1^; Jong Kwan Jun^15^ ^1^Seoul National University College of Medicine, Seoul, Seoul‐t'ukpyolsi; ^2^Dongguk University Ilsan Hospital, Goyang, Kyonggi‐do; ^3^Inha University Hospital, Inha University College of Medicine, Incheon, Inch'on‐jikhalsi; ^4^Seoul National University Bundang Hospital, Seoul National University College of Medicine, Bundang, Kyonggi‐do; ^5^Korea University College of Medicine, Seoul, Seoul‐t'ukpyolsi; ^6^University of Ulsan College of Medicine, Asan Medical Center, Seoul, Seoul‐t'ukpyolsi; ^7^101 Daehak‐ro, Jongno‐gu, Seoul, Seoul‐t'ukpyolsi; ^8^Seoul Metropolitan Government Seoul National University Boramae Medical Center, Seoul, Seoul‐t'ukpyolsi; ^9^College of Medicine, Gachon University, Incheon, Inch'on‐jikhalsi; ^10^Seoul St. Mary's Hospital, College of Medicine, The Catholic University of Korea, Seoul, Seoul‐t'ukpyolsi; ^11^Kangwon National University Hospital, School of Medicine, Kangwon National University, Chuncheon, Kangwon‐do; ^12^Chung‐Ang University Gwang‐Myeong Hospital, Chung‐Ang University College of Medicine, Gwang‐Myeong, Kyonggi‐do; ^13^Kyungpook National University Chilgok Hospital, Kyungpook National University, School of Medicine, Daegu, Ch'ungch'ong‐bukto; ^14^Seoul National University, Seoul, Seoul‐t'ukpyolsi; ^15^Ewha Womans University College of Medicine, Seoul, Seoul‐t'ukpyolsi
8:15 AM	2	**The Frequency and Severity of Complications after Previable PROM in Texas following SB8** Danna Ghafir^1^; **Emily Fahl^1^**; Nancy Ukoh^1^; Han‐Yang M. Chen^1^; Sean C. Blackwell^1^; Julie Gutierrez^2^; Irene A. Stafford^1^ ^1^McGovern Medical School at UTHealth Houston, Houston, TX; ^2^McGovern Medical School at The University of Texas Health Science Center at Houston (UTHealth), Houston, TX
8:30 AM	3	**Fetal fraction amplification enables accurate prenatal cell‐free DNA screening at 8 weeks gestation** **Lorraine Dugoff^1^**; Summer Pierson^2^; Jhett Bordwell^3^; Brent Mabey^2^; Arielle B. Dorch^3^; Alexander Gutin^2^; Dmitry Pruss^2^; Diana Iliev^2^; Dale Muzzey^2^; Katie Johansen Taber^4^ ^1^University of Pennsylvania, Philadelphia, PA; ^2^Myriad Genetics, Inc., Salt Lake City, UT; ^3^Myriad Genetics, Inc., South San Francisco, CA; ^4^Myriad Genetics, Inc., South San Fransisco, CA
8:45 AM	4	**AI Significantly Improves Detection of Prenatal Ultrasounds Suspicious for Major Congenital Heart Defects by OBGYN/MFMs** **Jennifer Lam‐Rachlin^1^**; Rajesh Punn^2^; Sarina K. Behera^3^; Miwa Geiger^1^; Matthias Lachaud^4^; Nadine David^5^; Sara Garmel^6^; Matthew K. Janssen^7^; Kendra Sylvester^8^; John Kennedy^9^; Jessica Spiegelman^1^; Mia A. Heiligenstein^10^; Nathan S. Fox^1^; Andrei Rebarber^1^; Greggory R. DeVore^11^; Carolyn M. Zelop^12^; Roger Bessis^13^; Marilyne Levy^14^; Bertrand Stos^14^; Malo De Boisredon^15^; Eric Askinazi^15^; Valentin Thorey^15^; Christophe Gardella^15^; Alisa Arunamata^2^ ^1^Icahn School of Medicine at Mount Sinai Hospital, New York, NY; ^2^Pediatrics ‐ Cardiology, Stanford University School of Medicine, Stanford, CA; ^3^Palo Alto Medical Foundation, Sutter Health, Palo Alto, CA; ^4^University of Grenoble Alpes, Grenoble, Rhone‐Alpes; ^5^Medical Training Center, Rouen, Haute‐Normandie; ^6^Michigan Perinatal Associates and Corewell Health, Dearborn, MI; ^7^University of Pennsylvania, Pittsburgh, PA; ^8^Perinatal Specialists of the Palm Beaches, West Palm Beach, FL; ^9^Wayne State University School of Medicine, Detroit, MI; ^10^Mount Sinai West, Astoria, NY; ^11^The Fetal Diagnostic Center of Pasadena, Pasadena, CA; ^12^Valley Health System, Paramus, NJ; ^13^Centre d'Echographie de l'Odéon, Paris, Ile‐de‐France; ^14^UE3C ‐ Unité d'explorations cardiologiques ‐ Cardiopathies Congénitales, Paris, Ile‐de‐France; ^15^BrightHeart, Paris, Ile‐de‐France
9:00 AM	5	**Atosiban versus placebo in threatened preterm birth (APOSTEL 8‐study): an international randomized controlled trial** **Larissa I. van der Windt^1^**; Job Klumper^1^; Ruben G. Duijnhoven^1^; Marjolein Kok^1^; Ben W. Mol^2^; Kate F. Walker^3^; Fionnuala M. M. McAuliffe^4^; Joris A. M. van der Post^5^; Carolien Roos^1^; Martijn A. Oudijk^1^ ^1^Amsterdam UMC, location University of Amsterdam, Amsterdam, Noord‐Holland; ^2^Monash University, Clayton, Victoria; ^3^Centre of Perinatal Research University of Nottingham, Queen's Medical Centre, Dublin, England; ^4^UCD Perinatal Research Centre, University College Dublin, Dublin 2, Dublin; ^5^Amsterdam UMC, location University of Amsterdam, Amsterdam, Noord‐Brabant
9:15 AM	6	**Prediction of severely small‐for‐gestational‐age infants using a novel cell‐free RNA model** Morten Rasmussen^1^; **Kara M. Rood^2^**; Aram Saravani^1^; Michal A. Elovitz^3^; Carrie Haverty^4^; Alison Moe^1^; Alison Cowan^1^; Arkady Khodursky^1^; Maneesh Jain^1^; Manfred Lee^1^; Thomas F. McElrath^5^; Elizabeth F. Sutton^6^; Arun Jeyabalan^7^; George R. Saade^8^; Antonio F. Saad^9^; Luis D. Pacheco^10^; Joseph R. Biggio, Jr^11^; Ebony B. Carter^12^; Antonina I. Frolova^13^; Esther Park‐Hwang^14^; Cynthia Gyamfi‐Bannerman^15^; Ai‐ris Y. Collier^16^; William A. Grobman^2^; Vincenzo Berghella^17^ ^1^Mirvie Inc., South San Francisco, CA; ^2^The Ohio State University, Columbus, OH; ^3^Icahn School of Medicine at Mount Sinai, New York, NY; ^4^Mirvie Inc., South San Fransisco, CA; ^5^Brigham Women's Hospital, Boston, MA; ^6^Woman's Hospital, Baton Rouge, LA; ^7^University of Pittsburgh School of Medicine, Pittsburgh, PA; ^8^Macon & Joan Brock Virginia Health Sciences Eastern Virginia Medical School at Old Dominion University, Norfolk, VA; ^9^Inova Health, Falls Church, VA; ^10^University of Texas Medical Branch, Galveston, TX; ^11^Ochsner Health, New Orleans, LA; ^12^University of North Carolina, Chapel Hill, NC; ^13^Washington University School of Medicine, St. Louis, MO; ^14^Multicare, Orlando, FL; ^15^University of California, San Diego, San Diego, CA; ^16^Beth Israel Deaconess Medical Center, Boston, MA; ^17^Sidney Kimmel Medical College of Thomas Jefferson University, Philadelphia, PA
9:30 AM	7	**Metformin paradoxically increases insulin resistance during pregnancy in a Rhesus macaque model** **Enrico R. Barrozo^1^**; Tyler Dean^2^; Claire E. Jensen^3^; Melissa A. Suter^1^; Maxim D. Seferovic^1^; Kristin Sauter^2^; Jacob E. Friedman^4^; Maureen Gannon^5^; Stephanie R. Wesoloski^6^; Carrie E. McCurdy^7^; Paul Kievit^2^; Kjersti M. Aagaard^8^ ^1^Baylor College of Medicine and Texas Children's Hospital, Houston, TX; ^2^Oregon National Primate Research Center, Beaverton, OR; ^3^University of North Carolina, Chapel Hill, NC; ^4^Harold Hamm Diabetes Center, University of Oklahoma Health Sciences Center, Oklahoma City, OK; ^5^Vanderbilt University Medical Center, Nashville, TN; ^6^University of Colorado, Anschutz Medical Campus, Aurora, CO; ^7^University of Oregon, Eugene, OR; ^8^Boston Children's Hospital, Division of Fetal Medicine and Surgery, Boston, MA; HCA Healthcare and HCA Healthcare Research Institute, Nashville, TN; HCA Texas Maternal Fetal Medicine, Houston, TX; Baylor College of Medicine and Texas Children's Hospital, Houston, TX, Boston, MA
9:45 AM	8	**INTER‐ACT interpregnancy and pregnancy lifestyle intervention for a healthy future: a randomized controlled trial** Yael Winter Shafran^1^; Hanne Van Uytsel^2^; Annick Bogaerts^2^; Lieveke Ameye^2^; **Roland Devlieger^3^** ^1^Kaplan Medical Center and REALIFE Research Group, Rehovot, HaMerkaz; ^2^REALIFE Research Group, KU Leuven, Leuven, Vlaams‐Brabant; ^3^University Hospital Leuven, Leuven, Vlaams‐Brabant

**Table:** 

Thursday, January 30, 2025 • 10:00 AM – 10:30 AM		
Time	Abstract	Late‐Breaking Abstract Presentations Session 1
10:00 AM	LB01	**CErebro Placental RAtio based management in reduced fetal movements; an international cluster‐randomized clinical trial (CEPRA)** **Laura A. Lens^1^**; Selina Posthuma^2^; Stefanie E. Damhuis^3^; Renée J. Burger, N/A^1^; Henk Groen^4^; Ruben G. Duijnhoven^5^; Sailesh Kumar^6^; Alexander E.P Heazell^7^; Asma Khalil^8^; Wessel Ganzevoort^9^; Sanne J. Gordijn^10^ ^1^Amsterdam UMC, Amsterdam, Noord‐Holland; ^2^Uiversity Medical Center Groningen, University Medical Center Groningen, Groningen; ^3^Amsterdam University Medical Centers, Amsterdam University Medical Centers, Noord‐Holland; ^4^University Medical Center of Groningen, University Medical Center of Groningen, Groningen; ^5^Amsterdam UMC, location University of Amsterdam, Amsterdam, Noord‐Holland; ^6^The University of Queensland, Brisbane, Queensland; ^7^The University of Manchester, Manchester, England; ^8^Fetal Medicine Unit, St George's Hospital, St George's University of London, Fetal Medicine Unit, St George's Hospital, S George's University of London, England; ^9^Amsterdam University Medical Centers, Amsterdam, Groningen; ^10^University Medical Center Groningen, Groningen, Groningen
10:15 AM	LB02	**iSEARCH RCT: Evaluating the effectiveness of maternal sildenafil to reduce complications related to intrapartum hypoxia** **Sailesh Kumar^1^**; Ben W. Mol^2^; William Tarnow‐Mordi^3^; Jon Hyett^4^; Nadia Badawi^4^; Annalene Seidler^5^; Helen Liley^6^; Emily Callander^7^; Vicky Flenady^6^; Sue Walker^8^; Rachel O'Connell^4^ ^1^The University of Queensland, Brisbane, Queensland; ^2^Monash University, Clayton, Victoria; ^3^The University of Sydney, Sydney, New South Wales; ^4^University of Sydney, Sydney, New South Wales; ^5^Medical Uni Rostock, Rostock, Mecklenburg‐Vorpommern; ^6^Mater Research Institute‐University of Queensland, Brisbane, Queensland; ^7^University Technology Sydney, Sydney, New South Wales; ^8^Department of Obstetrics, Gynaecology and Newborn Health, University of Melbourne, Melbourne, Victoria

**Table:** 

Thursday, January 30, 2025 • 1:30 PM ‐ 4:00 PM		
Time	Abstract	Oral Concurrent Session 1 ‐ Equity, Public Health, and Policy
1:30 PM	9	**Dysregulated gene expression in placentas with exposure to persistent maternal socioeconomic disadvantage** Greg E. Miller^1^; Lauren Keenan‐Devlin^2^; Alexa A. Freedman^1^; Renee Odom‐Konja^3^; Linda M. Ernst^3^; Steve Cole^4^; **Ann EB Borders^2^** ^1^Northwestern University, Chicago, IL; ^2^Endeavor Health, Evanston Hospital, Evanston, IL; ^3^Endeavor Health, Evanston, IL; ^4^University of California, Los Angeles, Los Angeles, CA
1:45 PM	10	**Association Between Maternal‐Fetal Medicine Physician Density and Adverse Pregnancy Outcomes** Tetsuya Kawakita; **Rula Atwani**; Lindsay S. Robbins; George R. Saade Macon & Joan Brock Virginia Health Sciences Eastern Virginia Medical School at Old Dominion University, Norfolk, VA
2:00 PM	11	**Black Maternal Morbidity and its Association with Systemic Racism** **Sebastian Z. Ramos**; Meghan I. Short; Erika F. Werner; Chloe E. Bird; Ndidiamaka Amutah‐Onukagha; Michael B. Siegel Tufts University School of Medicine, Boston, MA
2:15 PM	12	**Low‐income Birthing Individuals’ Need for and Satisfaction with Services in a One‐Year Postpartum Navigation Program** **Maya J. Daiter^1^**; Laura Diaz^1^; Hannah M. Green^1^; Ying Cheung^1^; Viridiana Carmona‐Barrera^1^; Brittney R. Williams^1^; Charlotte M. Niznik^1^; Joe M. Feinglass^1^; William A. Grobman^2^; Lynn M. Yee^1^ ^1^Northwestern University Feinberg School of Medicine, Chicago, IL; ^2^The Ohio State University, Columbus, OH
2:30 PM	13	**Impact of WIC Enrollment on Adverse Maternal and Neonatal Adverse Outcomes in Medicaid Populations** **Reetam Ganguli^1^**; Stephen Wagner^2^ ^1^Elythea, San Jose, CA; ^2^Beth Israel Deaconess Medical Center, Boston, MA
2:45 PM	14	**Homicide and Suicide: The Leading Cause of Maternal Death, and How Firearm Legislation Affects It** **Hooman A. Azad^1^**; Dana Goin^2^; Lisa Nathan^3^; Dena Goffman^1^; Uma M. Reddy^4^; Danielle Laraque‐Arena^2^; Mary E. D'Alton^1^ ^1^Columbia University Medical Center, New York, NY; ^2^Columbia University Mailman School of Public Health, New York, NY; ^3^Columbia University Irving Medical Center, New York, NY; ^4^Columbia University, New York, NY
3:00 PM	15	**Elimination of a threefold racial disparity in aspirin recommendation using integrated clinical decision support** **Melissa S. Wong^1^**; Rommy Coutelin Johnson^2^; Karla Gonzalez^1^; Ojiugo Onwumere^3^; Kristin Parrinella^4^; Camelita Thrift^3^; Samira Torna^1^; Matthew Wells^1^; Kimberly D. Gregory^3^ ^1^Cedars‐Sinai Medical Center, Los Angeles, CA; ^2^Cedars‐Sinai, Los Angeles, CA; ^3^Cedars Sinai Medical Center, Los Angeles, CA; ^4^Kaiser‐Permanente, Los Angeles, CA
3:15 PM	16	**Severe maternal morbidity differences by state Medicaid expansion and abortion coverage: national database analysis, 2014‐2022** **Ishana Shetty**; Mark C. Valentine; Mark Hoofnagle; Jennifer Reeves; David L. Eisenberg Washington University, St. Louis, MO
3:30 PM	17	**Elevated micro‐ and nanoplastics detected in preterm human placentae** **Enrico R. Barrozo^1^**; Marcus A. Garcia^2^; Michael D. Jochum, Jr.^1^; Rui Liu^2^; Alex Nihart^2^; Eliseo Castillo^2^; Eliane El Hayek^2^; Jorge Gonzalez‐Estrella^3^; Lori Showalter^1^; Cynthia Shope^1^; Melissa A. Suter^1^; Matthew J. Campen^2^; Kjersti M. Aagaard^4^ ^1^Baylor College of Medicine and Texas Children's Hospital, Houston, TX; ^2^University of New Mexico, Albuquerque, NM; ^3^Oklahoma State University, Stillwater, OK; ^4^Boston Children's Hospital, Division of Fetal Medicine and Surgery, Boston, MA; HCA Healthcare and HCA Healthcare Research Institute, Nashville, TN; HCA Texas Maternal Fetal Medicine, Houston, TX; Baylor College of Medicine and Texas Children's Hospital, Houston, TX, Boston, MA
3:45 PM	18	**Severe Maternal Morbidity among Asian and Pacific Islander Parturients in a Contemporary Northern California Cohort** **Shalila A. de Bourmont^1^**; Janet Alexander^2^; Baiyang Sun^2^; Erica P. Gunderson^2^; Mara Greenberg^1^ ^1^Kaiser‐Permanente Northern California, Oakland, CA; ^2^Kaiser‐Permanente Northern California, Pleasanton, CA

**Table:** 

Thursday, January 30, 2025 1:30 PM ‐ 4:00 PM		
Time	Abstract	Oral Concurrent Session 2 ‐ Clinical Obstetrics and Quality
1:30 PM	19	**Aberrant Fetal Growth in ARRIVE Trial** **Bonnie L. Hermann^1^**; Cassidy A. O'Sullivan^2^; Fabrizio Zullo^3^; Suneet Chauhan^4^; Hector M. Mendez‐Figueroa^5^ On behalf of the Eunice Kennedy Shriver National Institute of Child Health and Human Development Maternal‐Fetal Medicine Units (MFMU) Network ^1^UTHealth Houston, Houston, TX; ^2^Christiana Care Health System, Newark, DE; ^3^University of Rome La Sapienza, Rome, Lazio; ^4^Christiana Care, Newark, DE; ^5^McGovern Medical School at UTHealth, Houston, TX

**Table:** 

Thursday, January 30, 2025 • 1:30 PM ‐ 4:00 PM		
Time	Abstract	Oral Concurrent Session 2 ‐ Clinical Obstetrics and Quality, continued
1:45 PM	20	**Decreased Neuraxial Morphine Dose to Reduce Opioid Side Effects and use After Cesarean Delivery RCT** **Ayodeji Sanusi^1^**; Yumo Xue^2^; Kevin S. Shrestha^2^; Ayamo Oben^3^; Hanna Hussey^2^; Annalese Neuenschwander^2^; Michelle Tubinis^2^; Mark Powell^2^; Alan T. Tita^2^; Casey Brian^4^ ^1^Center for Women's Reproductive Health, University of Alabama at Birmingham, Birmingham, AL; ^2^University of Alabama at Birmingham, Birmingham, AL; ^3^Maternal Fetal Consultants of Houston, Houston, TX; ^4^West Virginia University, Morgantown, WV
2:00 PM	21	**Proportion of Time Spent in Category II Fetal Heart Tracing During Labor: Associated Adverse Outcomes** **Kristen A. Cagino^1^**; Rachel L. Wiley^2^; Aaron W. Roberts^3^; Claudia J. Ibarra^4^; Natalie L. Neff^5^; Kimen S. Balhotra^4^; Khalil M. Chahine^4^; Christina Cortes^4^; Shareen Patel^4^; Tala Ghorayeb^4^; Holly Flores^6^; Fabrizio Zullo^7^; Hector M. Mendez‐Figueroa^4^; Suneet Chauhan^8^ ^1^UT Houston, Houston, TX; ^2^University of California, San Diego, San Diego, CA; ^3^McGovern Medical School at UTHealth Houston, Houston, TX; ^4^McGovern Medical School at UTHealth, Houston, TX; ^5^McGovern Medical School at UT Health, Houston, TX; ^6^University of Texas Health Science Center, Houston, TX; ^7^University of Rome La Sapienza, Rome, Lazio; ^8^Christiana Care, Newark, DE
2:15 PM	22	**Tranexamic acid prophylaxis during cesarean delivery among patients with and without hypertension** **Adam K. Lewkowitz^1^**; Mariam Ayyash^2^ On behalf of the Eunice Kennedy Shriver National Institute of Child Health and Human Development Maternal‐Fetal Medicine Units (MFMU) Network ^1^Women & Infants Hospital of Rhode Island and Alpert Medical School of Brown University, Providence, RI; ^2^Columbia University Irving Medical Center, New York, NY
2:30 PM	23	**Preclinical Development of a Continuous Fetal Lactate Biosensor to Detect Acidosis** **Jonathan M. Morris^1^**; Arjun Kaushik^2^; Michael Challenor^2^; Lee J. Hubble^2^; Chaitya Shah^2^; Maureen Ross^2^; Ranjusha Rajagopalan^2^; Hassan Mahmood^2^; Allana Gurney^3^; Hayley Powell^3^; Jane Choi^3^; Helen Kershaw^3^; Gabrielle Musk^3^; Jane Pillow^3^ ^1^University of Sydney, New South Wales; ^2^VitalTrace Pty Ltd, Western Australia; ^3^University of Western Australia, Crawley, Western Australia
2:45 PM	24	**After Cesarean Time Interval to Exercise (ACTIVE) Trial: A Randomized Controlled Trial** **Brittany J. Roser^1^**; James Kinderknecht^2^; Brett Toresdahl^3^; Patricia Ladis^4^; Sonali Iyer^5^; Megan Savage^6^; Timothy Dekker^7^; Paul Christos^5^; Robin B. Kalish^8^ ^1^Stony Brook University Hospital, NY; ^2^Hospital for Special Surgery, New York, NY; ^3^University of Utah Health, Salt Lake City, UT; ^4^WiseBody Physical Therapists, New York, NY; ^5^Weill Cornell Medical College, New York, NY; ^6^Northwell Health at Lenox Hill, New York, NY; ^7^Ascension Medical Group, Rochester, MN; ^8^New York Presbyterian‐ Weill Cornell, New York, NY
3:00 PM	25	**Intrauterine Resuscitative Maneuvers for Management of Abnormal Fetal Heart Tracings: A Systematic Review and Meta‐Analysis** **Amanda J. Jones^1^**; Fabrizio Zullo^2^; Luis Sanchez‐Ramos^3^; Lauren C. Roby^1^; Stephanie Roth^4^; Suneet Chauhan^5^; Matthew K. Hoffman^1^; Anthony C. Sciscione^1^ ^1^Christiana Care Health System, Newark, DE; ^2^University of Rome La Sapienza, Rome, Lazio; ^3^University of Florida College of Medicine, Jacksonville, FL; ^4^Christiana Care Health Services, Newark, DE; ^5^Christiana Care, Newark, DE

**Table:** 

Thursday, January 30, 2025 • 1:30 PM ‐ 4:00 PM		
Time	Abstract	Oral Concurrent Session 2 ‐ Clinical Obstetrics and Quality, continued
3:15 PM	26	**Reducing Postpartum Hemorrhage: The Impact of a Standardized High‐Dose Oxytocin Protocol** **Evan J. Keil^1^**; Elizabeth J. Campbell^2^; Joanne M. Bailey^2^; David E. Arnolds^2^; Diana Peacor^2^; Molly J. Stout^2^; Jourdan E. Triebwasser^1^ ^1^University of Michigan, Ann Arbor, MI; ^2^University of Michigan Medical Center, Ann Arbor, MI
3:30 PM	27	**Hysterotomy using Barbed vs. Vicryl Suture for Scheduled Cesarean Delivery: A Randomized Controlled Trial** **Ayisha B. Buckley^1^**; Nicola F. Tavella^2^; Camila Cabrera^2^; Keisha S. Paul^2^; Mariah McKevitt^2^; Monica J. Patel^2^; Lauren A. Ferrara^2^; Jenny Tang^2^; Joanne Stone^3^; Calvin E. Lambert, Jr.^2^; Luciana A. Vieira^4^; Angela T. Bianco^2^ ^1^Weill Cornell Medical Center, New York, NY; ^2^Icahn School of Medicine at Mount Sinai, New York, NY; ^3^Mt. Sinai Medical Center, New York, NY; ^4^Stamford Hospital, Stamford, CT
3:45 PM	28	**Investigating selection bias in research using placental pathology samples** **Linda M. Ernst^1^**; Alexa A. Freedman^2^; Renee Odom‐Konja^1^; Lauren Keenan‐Devlin^3^; Greg E. Miller^2^; Ann EB Borders^3^ ^1^Endeavor Health, Evanston, IL; ^2^Northwestern University, Chicago, IL; ^3^Endeavor Health, Evanston Hospital, Evanston, IL

Thursday, January 30, 2025 • 1:30 PM ‐ 4:00 PM		
Time	Abstract	Oral Concurrent Session 3 ‐ Ultrasound and Genetics
1:30 PM	29	**Beyond the Fetus: Unveiling Maternal Health Risks Through Prenatal Genome Sequencing** **Brittany Arditi^1^**; Caitlin D. Baptiste^2^; Jessica L. Giordano^1^; Rachel Herz‐Roiphe^2^; Vaidehi Jobanputra^3^; Ronald J. Wapner^4^ ^1^Columbia University Irving Medical Center, New York, NY; ^2^Columbia University Medical Center, New York, NY; ^3^New York Genome Center, New York, NY; ^4^Columbia University, New York, NY
1:45 PM	30	**Artificial Intelligence System Accurately Detects Fetal Ultrasound Findings Suspicious For Major Congenital Heart Defects** **Carolyn M. Zelop^1^**; Jennifer Lam‐Rachlin^2^; Alisa Arunamata^3^; Rajesh Punn^3^; Sarina K. Behera^4^; Matthias Lachaud^5^; Nadine David^6^; Greggory R. DeVore^7^; Andrei Rebarber^2^; Nathan S. Fox^2^; Marjorie Gayanilo^2^; Sara Garmel^8^; Philippe Boukobza^9^; Pierre Uzan^10^; Hervé Joly^11^; Romain Girardot^12^; Laurence Cohen^13^; Marilyne Levy^14^; Bertrand Stos^14^; Malo De Boisredon^15^; Eric Askinazi^15^; Valentin Thorey^15^; Christophe Gardella^15^; Miwa Geiger^2^ ^1^Valley Health System, Paramus, NJ; ^2^Icahn School of Medicine at Mount Sinai Hospital, New York, NY; ^3^Pediatrics ‐ Cardiology, Stanford University School of Medicine, Stanford, CA; ^4^Palo Alto Medical Foundation, Sutter Health, Palo Alto, CA; ^5^University of Grenoble Alpes, Grenoble, Rhone‐Alpes; ^6^Medical Training Center, Rouen, Haute‐Normandie; ^7^The Fetal Diagnostic Center of Pasadena, Pasadena, CA; ^8^Michigan Perinatal Associates and Corewell Health, Dearborn, MI; ^9^CEDEF ‐ Centre Européen de Diagnostic et d'Exploration de la Femme, Le Chesnay, Ile‐de‐France; ^10^Groupe IMEF ‐ Imagerie Médicale de l'Est Francilien, Rosny‐sous‐Bois, Ile‐de‐France; ^11^CARPEDIOL ‐ Cardiologie Pédiatrique, fœtale et congénitale adulte de L'Ouest Lyonnais, Ecully, Rhone‐Alpes; ^12^Unité de Dépistage de Cardiopathies Foetales et Néonatales, Bordeaux, Aquitaine; ^13^ETCC ‐ Exploration et Traitement des Cardiopathies Congénitales, Massy, Ile‐de‐France; ^14^UE3C ‐ Unité d'explorations cardiologiques ‐ Cardiopathies Congénitales, Paris, Ile‐de‐France; ^15^BrightHeart, Paris, Ile‐de‐France
2:00 PM	31	**Effect of Home Ultrasound in Patients with Previous Late Pregnancy Loss‐ A Randomized Control Trial** **Liat Mor^1^**; Hagit Eisenberg^2^; Liliya Tamayev^3^; Daniel Tairy^1^; Ben Oren^3^; Yael Ganor Paz^1^; Michal Levy^3^; Eran Weiner^2^; Giulia Barda^2^ ^1^Edith Wolfson Medical Center, Holon, HaMerkaz; ^2^Wolfson Medical Center, Wolfson Medical Center, Tel Aviv; ^3^Edith Wolfson Medical center, Holon, HaMerkaz
2:15 PM	32	**Genetic diagnoses in a national cohort with non‐immune hydrops and other fetal effusions** **Natalie B. Gulrajani^1^**; Billie R. Lianoglou^2^; Katie Tick^3^; Nuriye Sahin‐Hodoglugil^3^; Ugur Hodoglugil^4^; Patrick Devine^4^; Jessica Van Ziffle^4^; Mary E. Norton^2^; Teresa N. Sparks^3^ ^1^University of California, San Francisco, School of Medicine, San Francisco, CA; ^2^Center for Maternal‐Fetal Precision Medicine, University of California San Francisco, San Francisco, CA; ^3^University of California, San Francisco, San Francisco, CA; ^4^Genomic Medicine Laboratory, University of California, San Francisco, San Francisco, CA
2:30 PM	33	**Genomewide monogenic Non Invasive Prenatal Genetic Screening based on maternal cfDNA** Dolev Rahat^1^; Ravit Mesika^1^; Lilach Schneor^1^; **Noa Liscovitch‐Brauer^2^**; Tom Rabinowitz^1^; Noam Shomron^1^; Reut Tomashov Matar^3^; Lina Basel‐Salmon^3^ ^1^Identifai Genetics, Tel Aviv, Israel; ^2^Identifai Genetics, Tel Aviv; ^3^Raphael Recanati Genetic Institute, Rabin Medical Center, Beilinson Hospital, Tel Aviv
2:45 PM	34	**AI Assistance Enhances Physician Performance in Identifying Congenital Malformations** Clémentine Morisset^1^; Frédéric Logé‐Munerel^1^; **Celia Amabile^1^**; Louis Chouinard^1^; Vianney Debavelaere^1^; Remi Besson^1^; Nikola Matevski^1^; Guillaume Corda^1^; Julien Stirnemann^2^; Yves Ville^3^; Yinka Oyelese^4^; Andrew Combs^5^ ^1^Sonio, Paris, Ile‐de‐France; ^2^Hôpital Necker Enfants Malades, Paris, Ile‐de‐France; ^3^University and Necker‐Enfants Malades Hospital, Paris, Ile‐de‐France; ^4^Beth Israel Deaconess Medical Center/Harvard Medical School, Boston, MA; ^5^Pediatrix Medical Group, Sunrise, FL
3:00 PM	35	**Exploring Gastroschisis Patterns and Pesticide Use in California's Central Valley: A population‐based cohort Study** **Alejandro Perez^1^**; Bharti Garg^2^; Hope Delgado^3^; Joshua F. Robinson^4^; Aaron B. Caughey^2^; Stephanie L. Gaw^4^ ^1^University of California, San Francisco, Kerman, CA; ^2^Oregon Health & Science University, Portland, OR; ^3^University of California, Berkeley, Berkeley, CA; ^4^University of California, San Francisco, San Francisco, CA
3:15 PM	36	**AI software demonstrates strong performance for screening of Tetralogy of Fallot and Truncus Arteriosus Communis** **Remi Besson^1^**; Nicolas Fries^2^; Julien Stirnemann^3^; Yves Ville^4^; Guy Vaksmann^5^ ^1^Sonio, Paris, Ile‐de‐France; ^2^Imagyn'Echo, Imagyn'echo Montpellier, Languedoc‐Roussillon; ^3^Hôpital Necker Enfants Malades, Paris, Ile‐de‐France; ^4^University and Necker‐Enfants Malades Hospital, Paris, Ile‐de‐France; ^5^Cabinet Vendôme, Lille, Nord‐Pas‐de‐Calais
3:30 PM	37	**Genetic Etiologies of Bilateral Renal Agenesis** **Bobby Brar^1^**; Robert Weatherford^2^; Carol Nowlen^1^; Katelynn Sagaser^3^; Ahmet A. Baschat^1^; Karin Blakemore^1^; Jena L. Miller^1^; Angie C. Jelin^1^ ^1^Johns Hopkins Medicine, Baltimore, MD; ^2^Massachusetts General Hospital, Boston, MA; ^3^23andMe, South San Fransisco, CA
3:45 PM	38	**Fully Quantitative Cervical Remodeling: Inter‐pregnancy interval shows differences in biomechanical characteristics of the cervix** **Sarah M. Dwyer^1^**; LeAnn A. Louis^1^; Methodius G. Tuuli^2^; Adam K. Lewkowitz^2^; Julie Tumbarello^1^; Emily Dively, BSN^3^; Madeline Felske^1^; Wendy Sparks^1^; Giselle Kolenic^4^; Peinan Zhao^5^; Molly J. Stout^6^ ^1^University of Michigan Hospital, Ann Arbor, MI; ^2^Women & Infants Hospital of Rhode Island and Alpert Medical School of Brown University, Providence, RI; ^3^Washington University School of Medicine, St. Louis, MO; ^4^University of Michigan, Ann Arbor, MI; ^5^Washington University, St. Louis, MO; ^6^University of Michigan Medical Center, Ann Arbor, MI

**Table:** 

Friday, January 31, 2025 • 8:00 AM ‐ 10:00 AM		
Time	Abstract	Oral Plenary Session 2 (Fellows Plenary)
8:00 AM	39	**Methylergonovine to Decrease Blood Loss During Cesarean Delivery in Twins: A Triple‐Blinded Placebo‐Controlled Randomized Trial** **Helen B. Gomez Slagle^1^**; Shai Bejerano^2^; Russell S. Miller^2^; Dena Goffman^2^; Mary E. D'Alton^2^; Mirella Mourad^2^ ^1^Columbia University Irving Medical Center, New York, NY; ^2^Columbia University Medical Center, New York, NY
8:15 AM	40	**Management of postpartum preeclampsia and hypertensive disorders (MOPP): Postpartum tight versus standard blood pressure control** **Emily B. Rosenfeld^1^**; Deepika Sagaram^1^; Rachel Lee^1^; Ernani Sadural^2^; Richard C. Miller^2^; Ruby Lin^1^; Deshae Jenkins^1^; Kristin Blackledge^3^; Ivana Nikodijevic^4^; Alex Rizzo^2^; Vanessa Martinez^1^; Emily E. Daggett^5^; Olivia McGeough^1^; Cande V. Ananth^1^; Todd J. Rosen^1^ ^1^Rutgers Robert Wood Johnson Medical School, New Brunswick, NJ; ^2^Cooperman Barnabas Medical Center, RWJBarnabas Health, Livingston, NJ; ^3^Rutgers New Jersey Medical School and Cooperman Barnabas Medical Center, Livingston, NJ; ^4^New Jersey Medical School, Newark, NJ; ^5^Rutgers Robert Wood Johnson Medical School, Edison, NJ
8:30 AM	41	**Impact of living in a food desert on prenatal macro‐ and micronutrient intake** **Noor K. Al‐Shibli^1^**; Anne L. Dunlop^2^; Suchitra Chandrasekaran^3^ ^1^Emory University, School of Medicine, Atlanta, GA; ^2^Emory University School of Medicine, Atlanta, GA; ^3^Emory University, Atlanta, GA
8:45 AM	42	**Small for gestational age prediction using unsupervised machine learning of first trimester fetal cardiac parameters** **Rebecca Horgan^1^**; Elena Sinkovskaya^1^; Erkan Kalafat^2^; George R. Saade^1^; Alfred Abuhamad^3^ ^1^Macon & Joan Brock Virginia Health Sciences Eastern Virginia Medical School at Old Dominion University, Norfolk, VA; ^2^Koc University Hospital, Istanbul, Istanbul; ^3^Eastern Virginia Medical School, Norfolk, VA
9:00 AM	43	**Pravastatin dose‐range and pharmacokinetic study in a pregnant rat model** **Xiao‐Yu Wang^1^**; Sydney Lammers^2^; Jennifer Reno‐Graber^3^; Frederick Reno^3^; Alan Hoberman^4^; George R. Saade^5^; Monica Longo^6^; Victoria L. Pemberton^7^; Ronald J. Wapner^8^; Nicole Abbott^1^; Zhiliang Xie^1^; Joo Young Na^1^; Mitch A. Phelps^1^; Maged M. Costantine^1^ ^1^The Ohio State University, Columbus, OH; ^2^The Ohio State University, College of Medicine, Columbus, OH; ^3^Reno & Associates, FL; ^4^Charles River Laboratories, PA; ^5^Macon & Joan Brock Virginia Health Sciences Eastern Virginia Medical School at Old Dominion University, Norfolk, VA; ^6^National Institute of Child Health and Human Development, Bethesda, MD; ^7^National Heart, Lung, and Blood Institute, Bethesda, MD; ^8^Columbia University, New York, NY
9:15 AM	44	**Patterns of perceived and everyday stress, resilience, and adverse placentally mediated outcomes** **Maura Jones Pullins**; Sarah Heerboth; Joy McNeal; Ashlyn Tolbert; Annie Dude; Johanna Quist‐Nelson; Rebecca Fry; Tracy A. Manuck University of North Carolina, Chapel Hill, NC
9:30 AM	45	**Microfluidic Device Successfully Replaces Traditional Models of Pregnancy Associated Drug Pharmacokinetic Studies** **Ana Collins‐Smith**; Ananth Kammala; Lauren Richardson; Xiao‐ming Wang; Ramkumar Menon University of Texas Medical Branch, Galveston, TX
9:45 AM	46	**Continuous Glucose Monitoring for Gestational Diabetes Diagnosis: A Comparative Effectiveness Randomized Control Trial (PRECISE)** **Sarah A. Nazeer^1^**; Joycelyn A. Corntwaithe, RD, CDE^1^; Rafael Bravo Santos^1^; Claudia Pedroza^1^; Sean C. Blackwell^2^; Suneet Chauhan^3^; Farah H. Amro^2^; Ghamar Bitar^2^; Jon Tyson^1^; Baha M. Sibai^2^; Michal Fishel Bartal^4^ ^1^The University of Texas Health Science Center at Houston (UTHealth), Houston, TX; ^2^McGovern Medical School at UTHealth Houston, Houston, TX; ^3^Christiana Care, Newark, DE; ^4^UTH Houston & Sheba Medical Center Israel, Houston, TX

**Table:** 

Friday, January 31, 2025 • 10:00 AM – 10:30 AM		
Time	Abstract	Late‐Breaking Abstract Presentations Session 2
10:00 AM	LB03	**Single‐dose intravenous iron versus oral iron for maternal iron deficiency anemia: a randomized controlled trial** Richard Derman^1^; Mrutyunjaya Bellad^2^; Manjunath Somannavar^2^; Sudhir Bhandari^3^; Sudhir Mehta^3^; Seema Mehta^3^; Dharmesh Sharma^3^; Yogesh Kumar^4^; Umesh Charantimath^4^; Amaresh Patil^5^; Ashalata Mallapur^6^; Umesh Ramdurg^6^; Radha Sangavi^7^; Praveen Patil^7^; Subarana Roy^8^; Phaniraj Vastrad^9^; Chander Shekhar^10^; Benjamin Leiby^10^; Rebecca Hartman^10^; Michael Georgieff^11^; Stephen Mennemeyer^10^; Zubair H. Aghai^12^; Simal Thind^10^; **Rupsa C. Boelig^13^** ^1^Jefferson Health, Philadelphia, PA; ^2^KLE Academy of Higher Education and Research's J N Medical College,, Belagavi, Karnataka; ^3^Sawai Man Singh Medical College, Jaipur, Rajasthan; ^4^KLE Academy of Higher Education and Research's J N Medical College,, KLE Academy of Higher Education and Research's J N Medical College,, Karnataka; ^5^KLE Academy of Higher Education and Research's J N Medical College, Belagavi, Karnataka; ^6^S Nijalingappa Medical and HSK Hospital and Research Center, Bagalkote, Karnataka; ^7^Raichur Institute of Medical Sciences, Raichur, Karnataka; ^8^ICMR – National Institute of Traditional Medicine, Belagavi, Karnataka; ^9^Model Rural Health Research Unit, Sirwar, Karnataka; ^10^Thomas Jefferson University, Philadelphia, PA; ^11^University of Minnesota, Minneapolis, MN; ^12^Sidney Kimmel Medical College at Thomas Jefferson University, Philadelphia, PA; ^13^Sidney Kimmel Medical College, Thomas Jefferson University, Philadelphia, PA
10:15 AM	LB04	**Neonatal impact of prematurity risk biomarker screening with targeted interventions: A multicenter randomized controlled trial** Brian K. Iriye^1^; John O'Brien^2^; Christopher S. Ennen^3^; Perry Barrilleaux^4^; Jill Berkin^5^; Anna Palatnik^6^; Moeun Son^7^; Cynthia Gyamfi‐Bannerman^8^; Mollie McDonald^9^; Glenn Markenson^10^; Anthony C. Sciscione^11^; Joseph R. Biggio, Jr^12^; Samuel Wolf^13^; Scott Sullivan^14^; Michael Walker^15^; Babak Shahbaba^16^; Stephen P. Pound^17^ ^1^Hera Womens Health, Las Vegas, NV; ^2^University of Kentucky, Lexington, KY; ^3^University of Virginia School of Medicine, Charlottesville, VA; ^4^Ochsner Louisiana State University Health Shreveport, Shreveport, LA; ^5^Icahn School of Medicine at Mount Sinai, New York, NY; ^6^Medical College of Wisconsin, Milwaukee, WI; ^7^Weill Cornell Medicine, New York, NY; ^8^University of California, San Diego, San Diego, CA; ^9^Austin Maternal‐Fetal Medicine, Austin, TX; ^10^Boston Medical Center, Boston, MA; ^11^Christiana Care Health System, Newark, DE; ^12^Ochsner Health, New Orleans, LA; ^13^Emerald Coast OBGYN Clinical Research, Panama City, FL; ^14^Inova Fairfax Medical Campus, Fairfax, VA; ^15^Walker Bioscience, Carlsbad, CA; ^16^University of California, Irvine, Irvine, CA; ^17^Virginia Physicians for Women, North Chesterfield, VA

Friday, January 31, 2025 • 1:30 PM ‐ 4:00 PM		
Time	Abstract	Oral Concurrent Session 4 ‐ Basic and Translational Science
1:30 PM	47	**Novel Neural Extracellular Vesicle miRNA Biomarkers Identify Suboptimal Response to Cooling in Neonatal Encephalopathy** **Sarah T. Mehl^1^**; Baharan Fekry^1^; Lierni Ugartemendia^1^; Dhanashree Rajderkar^2^; Nikolay Bliznyuk^2^; Shaveka Gaskins^2^; Michael D. Weiss^3^; Laura Goetzl^1^ ^1^McGovern Medical School at UTHealth Houston, Houston, TX; ^2^University of Florida, Gainesville, FL; ^3^University of Florida College of Medicine, Gainesville, FL
1:45 PM	48	**3D‐printed humanized feto‐maternal interface tests exosomal delivery of anti‐inflammatory Interleukin‐10 (IL‐10) to reduce infection‐associated inflammation** Leah Saylor^1^; Rahul Cherukuri^2^; Ananth Kammala^1^; Marc Ferrer^3^; Cristina Antich Acedo^3^; Arum Han^2^; Lauren Richardson^1^; **Ramkumar Menon^1^** ^1^University of Texas Medical Branch, Galveston, TX; ^2^Texas A&M University, College Station, TX; ^3^National Center for Advancing Translational Sciences, National Institute of Sciences, Bethesda, MD
2:00 PM	49	**Decoy peptide to (pro)renin receptor as a potential therapeutic target for preeclampsia: a translational study** **Michelle Harris^1^**; Ahmed F. Pantho^2^; Kelsey R. Kelso^1^; Jessica C. Ehrig^3^; Ram R. Kalagiri^1^; Niraj Vora^1^; Thomas J. Kuehl^2^; Steven R. Lindheim^1^; Mohammad N. Uddin^4^ ^1^Baylor Scott & White Health Temple Medical Center, Temple, TX; ^2^Artemis Biotechnologies LLC, Temple, TX; ^3^Baylor Scott and White Health, Temple, TX; ^4^Baylor Scott & White Medical Center and Texas A&M School of Medicine, Temple, TX

**Table:** 

Friday, January 31, 2025 • 1:30 PM ‐ 4:00 PM		
Time	Abstract	Oral Concurrent Session 4 ‐ Basic and Translational Science, continued
2:15 PM	50	**Longitudinal changes in epigenetic aging in pregnant versus non‐pregnant people: a prospective cohort study** **Danielle M. Panelli**; Nicole Gladish; Nicola C. Perlman; Andres Cardenas; Katherine Bianco Stanford University, Palo Alto, CA
2:30 PM	51	**Lactobacillus crispatus mitigates Group B Streptococcus‐induced inflammation in the cervicovaginal epithelium** Briana Ferguson; Aaron Loder; Lauren Anton; **Kristin D. Gerson** University of Pennsylvania Perelman School of Medicine, Philadelphia, PA
2:45 PM	52	**Expression of CD300E in human myometrium and its impact on parturition** **Meera M. Thakkar^1^**; William E. Ackerman, IV^1^; Guomao Zhao^1^; Zoe B. Strong^1^; Elizabeth Feoktistov^2^; Ekaterina Snegovskikh^2^; Catalin S. Buhimschi^1^; Irina A. Buhimschi^1^ ^1^University of Illinois at Chicago, College of Medicine, Chicago, IL; ^2^University of Illinois at Chicago, Chicago, IL
3:00 PM	53	**Personalized models of fetal brain development as biomarkers of offspring neurodevelopmental outcomes after maternal SARS‐CoV‐2** Lydia L. Shook; Liam T. McCrea; Olyvia Jasset; Steven D. Sheridan; Roy H. Perlis; **Andrea G. Edlow** Massachusetts General Hospital, Boston, MA
3:15 PM	54	**The Placental Vascular Transcriptome in Pregnancies Resulting in Low vs. Normal Birth Weight Neonates** **Samantha Carson^1^**; Morgan E. Wasickanin^1^; Emily Sheikh^1^; Hillary Kinsman^1^; Lydia Bettridge^1^; Katherine E. Free^1^; Jennifer Damicis^1^; Robert Walton^1^; Peter Napolitano^2^; Nicholas Ieronimakis^1^ ^1^Madigan Army Medical Center, Tacoma, WA; ^2^University of Washington, Seattle, WA
3:30 PM	55	**Commonly Used Non‐Steroidal Anti‐Inflammatory Drug (NSAIDs) in Obstetrics & Associated Placental Cytotoxicity** **Ana Collins‐Smith** University of Texas Medical Branch, Galveston, TX
3:45 PM	56	**Evaluating Semaglutide in Human Fetal Neural Stem Cells: Could Early Exposure Impact Neurodevelopment?** **Morgan E. Wasickanin^1^**; Samantha Carson^1^; Hillary Kinsman^1^; Katherine E. Free^1^; Jennifer Damicis^1^; Emily Sheikh^1^; Robert Walton^1^; Irina Burd^2^; Peter Napolitano^3^; Nicholas Ieronimakis^1^ ^1^Madigan Army Medical Center, Tacoma, WA; ^2^University of Maryland, Baltimore, MD; ^3^University of Washington, Seattle, WA
1:30 PM	57	**Myo‐inositol Supplementation to Prevent Pregnancy Complications in Polycystic Ovary Syndrome: a Randomized Controlled Trial** **Anne W.T. Van Der Wel^1^**; Rebekka Bout‐Rebel^2^; Chryselle M.C. Frank^3^; Ruben G. Duijnhoven^1^; Bo E. Van Bree^4^; Olivier Valkenburg^4^; Salwan Al‐Nasiry^4^; Robbert H.F. van Oppenraaij^5^; Tatjana E. Vogelvang^6^; Michelle E.M.H Westerhuis^7^; Jan Peter de Bruin^8^; Hedwig P. van de Nieuwenhof^8^; Susanne C.J.P. Gielen^9^; Myrthe L. Bandell^10^; Mireille N. Bekker^11^; Maurice G.A.J. Wouters^1^; Velja Mijatovic^1^; Arie Franx^2^; Cornelis B. Lambalk^1^; Frank J.M. Broekmans^11^; Rebecca C. Painter^1^; Bart C.J.M. Fauser^12^; Joop S.E. Laven^2^; Bas B. van Rijn^13^; MYPP Investigator Group^2^ ^1^Amsterdam UMC, location University of Amsterdam, Amsterdam, Noord‐Holland; ^2^Erasmus University Medical Center, Zuid‐Holland; ^3^St. Antonius Ziekenhuis, Utrecht; ^4^Maastricht UMC+, Limburg; ^5^Maasstad Ziekenhuis, Zuid‐Holland; ^6^Het Diakonessenhuis, Utrecht; ^7^Catharina Ziekenhuis, Noord‐Brabant; ^8^Jeroen Bosch Ziekenhuis, Noord‐Brabant; ^9^Franciscus, Zuid‐Holland; ^10^Albert Schweitzer ziekenhuis, Zuid‐Holland; ^11^University Medical Center Utrecht, Utrecht; ^12^University of Utrecht and University Medical Center Utrecht, Utrecht; ^13^Eindhoven University of Technology, Noord‐Brabant

Friday, January 31, 2025 • 1:30 PM ‐ 4:00 PM		
Time	Abstract	Oral Concurrent Session 5 ‐ Hypertension
1:45 PM	58	**Nifedipine versus Labetalol for Treatment of Postpartum Hypertension: A Randomized Controlled Trial** **Todd R. Lovgren^1^**; Ruofan Yao^2^; Brendan D. Connealy^3^; Robert Bonebrake^1^; Hemant Satpathy^1^; Emily Patel^1^; Matthew Brady^1^; Joshua Dahlke^4^ ^1^Nebraska Methodist Health System, Elkhorn, NE; ^2^Loma Linda University Health, Loma Linda, CA; ^3^Methodist Women's Hospital, Omaha, NE; ^4^Nebraska Methodist Hospital, Elkhorn, NE
2:00 PM	59	**Defining postpartum cardiovascular physiology: Blood pressure trajectories in patients at risk for new‐onset postpartum hypertension** **Ukachi N. Emeruwa^1^**; Minhazur R. Sarker^1^; Elizabeth Nicole Teal^1^; Marni B. Jacobs^2^; Louise C. Laurent^3^; Natalie A. Bello^4^; Timothy Wen^5^; Russell S. Miller^6^; Cynthia Gyamfi‐Bannerman^1^ ^1^University of California, San Diego, San Diego, CA; ^2^University of California, San Diego Health, San Diego, CA; ^3^University of California, San Diego Medical Center, La Jolla, CA; ^4^Cedars Sinai Medical Center, Los Angeles, CA; ^5^University of California, San Diego, Irvine, CA; ^6^Columbia University Medical Center, New York, NY
2:15 PM	60	**Severe Systolic Hypertension and Adverse Maternal Outcomes** **Yossi Bart^1^**; Hector M. Mendez‐Figueroa^2^; Farah H. Amro^1^; Baha M. Sibai^1^ ^1^McGovern Medical School at UTHealth Houston, Houston, TX; ^2^McGovern Medical School at UTHealth, Houston, TX
2:30 PM	61	**Postpartum Diuretics for Hypertensive Disorders of Pregnancy: A Systematic Review and Meta‐Analysis** **Emma Trawick Roberts^1^**; Elana Jaffe^1^; Johanna Quist‐Nelson^1^; Suneet Chauhan^2^; Baha M. Sibai^3^; Michal Fishel Bartal^4^ ^1^University of North Carolina, Chapel Hill, NC; ^2^Christiana Care, Newark, DE; ^3^McGovern Medical School at UTHealth Houston, Houston, TX; ^4^UTH Houston & Sheba Medical Center Israel, Houston, TX
2:45 PM	62	**Association of Preeclampsia with Long‐Term Risk of Neurodegenerative Disorders** **Maria Bazan^1^**; Ai‐ris Y. Collier^2^; Tina Yi Jin Hsieh^2^; James Cheng‐Chung Wei^3^ ^1^Beth Israel Deaconess Medical Center, Brighton, MA; ^2^Beth Israel Deaconess Medical Center, Boston, MA; ^3^Chung‐Shan Medical School, Taichung, Taichung
3:00 PM	63	**Comprehensive blood pressure management to improve early postpartum blood pressure parameters: a randomized controlled trial** **Alexandra JD Phelps^1^**; Loveis Jackson^1^; Lilly He^1^; Ashanti E. Griggs‐Cooks^1^; Neha Dudipala^2^; Etoi Garrison^1^; Kathryn J. Lindley^1^; Soha S. Patel^1^; Julia C. Phillippi^3^; Megan Webb^1^; Sarah S. Osmundson^1^ ^1^Vanderbilt University Medical Center, Nashville, TN; ^2^Ohio State University College of Medicine, Columbus, OH; ^3^Vanderbilt University School of Nursing, Nashville, TN
3:15 PM	64	**Hofbauer cells may mediate PE‐associated placental dysfunction via APOE synthesis** **Bani Medegan Fagla**; Cielo Dela Rosa; Savita Sundar; Aswathi Jayaram; Erykah Walton; Jason York; Leon M. Tai; Guomao Zhao; Irina A. Buhimschi University of Illinois at Chicago, College of Medicine, Chicago, IL
3:30 PM	65	**Impact on Severe Maternal Morbidity following Statewide Improvements in Hypertensive Disorders of Pregnancy Care Processes** **Patrick Schneider^1^**; Allison Stevens^2^; Alyssa Antonini^2^; Michelle Menegay^3^; Allison Lorenz^3^; Stephen Afflito^3^; Rashelle Ghanem^4^; Ryan Everett^5^; Justin R. Lappen^6^ ^1^The Ohio State University, Wexner Medical Center, Columbus, OH; ^2^Government Resources Center for the Ohio Colleges of Medicine, Columbus, OH; ^3^Ohio Colleges of Medicine, Government Resource Center, Columbus, OH; ^4^Ohio Department of Children and Youth, Columbus, OH; ^5^Ohio Hospital Association, Columbus, OH; ^6^Cleveland Clinic Foundation, Cleveland, OH
3:45 PM	66	**Adverse outcomes during delivery hospitalizations among patients with an intellectual or developmental disability diagnosis** **Manasa G. Rao^1^**; Timothy Wen^2^; Mary E. D'Alton^1^; Alexander M. Friedman^3^; Noelia Zork^3^ ^1^Columbia University Medical Center, New York, NY; ^2^University of California, San Diego, Irvine, CA; ^3^Columbia University Irving Medical Center, New York, NY

Friday, January 31, 2025 • 1:30 PM ‐ 4:00 PM		
Time	Abstract	Oral Concurrent Session 6 ‐ Prematurity and Newborn
1:30 PM	67	**Azithromycin to prevent stillbirths and infant deaths in Mali: A 2x2 factorial placebo‐controlled randomized trial** **Karen Kotloff^1^**; Amanda J. Driscoll^1^; Fadima Haidara^2^; Lawrence Moulton^3^; Jason Bailey^1^; Ousmane Samake^2^; Tiecoura Bocoum^2^; Jane Juma^2^; Awa Traore^2^; Mamadou Diallo^2^; Collins Okello^2^; Uma Onwuchekwa^2^; Mamoudou Kodio^2^; Yuji Chen^1^; Emily Deichsel^1^; Matthew Finholt‐Daniel^4^; Robert L. Goldenberg^5^; Fleesie Hubbard^1^; Rebecca Maguire^1^; Melissa Page^4^; David Plotner^4^; Milagritos Tapia^1^; Dilruba Nasrin^1^; Samba Sow^2^ ^1^University of Maryland, School of Medicine, Baltimore, MD; ^2^Centre pour le Développement des Vaccins, Mali, Bamako, Bamako; ^3^Johns Hopkins, Bloomberg School of Public Health, Baltimore, MD; ^4^RTI International, Durham, NC; ^5^Columbia University School of Medicine, New York, NY
1:45 PM	68	**The effect of metformin in women who received betamethasone on maternal hyperglycemia and neonatal hypoglycemia** **Enav Yefet^1^**; Manal Massalha^2^; Gil Talmon^1^; Aminet Labay^1^; Marian matanis^1^; Erez Sleman^1^; Rima nassra^1^; Maya Frank Wolf^3^; Inshirah Sgayer^4^; Lior Lowenstein^4^; Zohar Nachum^2^ ^1^Tzafon Medical Center, Poriya, HaZafon; ^2^Emak Medical Center, Afula, HaZafon; ^3^Galilee Medical Center, Naharyia, HaZafon; ^4^Galilee Medical center, Nahariya, HaZafon
2:00 PM	69	**Fully Quantitative Cervical Remodeling: Race Group Differences Responsibilities and Cautions** **LeAnn A. Louis^1^**; Sarah M. Dwyer^1^; Methodius G. Tuuli^2^; Adam K. Lewkowitz^2^; Julie Tumbarello^1^; Emily Dively, BSN^3^; Madeline Felske^1^; Wendy Sparks^1^; Anita M. Malone^1^; Peinan Zhao^4^; Molly J. Stout^5^ ^1^University of Michigan Hospital, Ann Arbor, MI; ^2^Women & Infants Hospital of Rhode Island and Alpert Medical School of Brown University, Providence, RI; ^3^Washington University School of Medicine, St. Louis, MO; ^4^Washington University, St. Louis, MO; ^5^University of Michigan Medical Center, Ann Arbor, MI
2:15 PM	70	**Neonatal outcomes after Caesarean versus vaginal birth: population‐based cohort study of extremely preterm breech singletons** **Yanchen Wang^1^**; Pasqualina Santaguida^1^; Sameer Parpia^1^; Fabiana Bacchini^2^; Prakeshkumar S. Shah^3^; Kellie Murphy^3^; K. S. Joseph^4^; Sandesh Shivananda^5^; Sarah D. McDonald^1^ ^1^McMaster University, Hamilton, ON; ^2^Canadian Premature Babies Foundation, Toronto, ON; ^3^University of Toronto, Toronto, ON; ^4^School of Population and Public Health, University of British Columbia, Vancouver, BC; ^5^University of British Columbia, Vancouver, BC
2:30 PM	71	**The role of cervical elastography for the prediction of spontaneous preterm birth** **Anne‐Sophie Lafortune^1^**; Louise Ghesquiere^2^; Paul Guerby^3^; Marie‐Laurence Côté^1^; Genevieve Marcoux^4^; Annie Beaudoin^4^; Emmanuel Bujold^1^ ^1^Université Laval, PQ; ^2^Université de Lille, Lille, Nord‐Pas‐de‐Calais; ^3^Université de Toulouse, Midi‐Pyrenees; ^4^CHU de Québec, CHU de Québec, PQ
2:45 PM	72	**Corticosteroids and Neonatal Hypoglycemia among Pregnant Individuals with Diabetes: Effect of Gestational Age at Delivery** **Ruby Lin**; Cande V. Ananth; Todd J. Rosen On behalf of the MOMPOD Consortium Rutgers Robert Wood Johnson Medical School, New Brunswick, NJ
3:00 PM	73	**Pro‐inflammatory vaginal cytokines, early PTB, and infectious morbidity in patients with asymptomatic cervical shortening** **Tracy A. Manuck** On behalf of the Eunice Kennedy Shriver National Institute of Child Health and Human Development Maternal‐Fetal Medicine Units (MFMU) Network University of North Carolina, Chapel Hill, NC

Friday, January 31, 2025 • 1:30 PM ‐ 4:00 PM		
Time	Abstract	Oral Concurrent Session 6 ‐ Prematurity and Newborn, continued
3:15 PM	74	**Comparison of Polyester Fiber versus Polypropylene Suture Materials in Physical‐Exam Indicated Transvaginal Cerclages** Daniel J. Martingano^1^; Amanda F. Francis Oladipo^2^; **Marwah Al‐Dulaimi^3^**; Sandra Kumwong^4^; Andrea Ouyang^5^; Lauren Cue^6^; Ashley Nguyen^3^; Shailini Singh^7^; Alexander Ulfers^8^; Mark Rebolos^3^; Kristin Cohen^9^; Donald Morrish^3^; Iffath A. Hoskins^10^; Francis X. Martingano^11^ ^1^St. John's Episcopal Hospital‐South Shore and William Carey University College of Osteopathic Medicine, Far Rockaway, NY; ^2^Hackensack University Medical Center, Hackensack, NJ; ^3^St. John's Episcopal Hospital‐South Shore, Far Rockaway, NY; ^4^Touro College of Osteopathic Medicine‐Harlem Campus, New York, NY; ^5^William Carey University, Hattiesburg, MS; ^6^Rutgers University and the Jersey City Medical Center, Jersey City, NJ; ^7^AtlantiCare Regional Medical Center, Pamona, NJ; ^8^Walter Reed National Military Medical Center, Bethesda, MD; ^9^RWJBarnabas Health ‐ Trinitas Regional Medical Center, Elizabeth, NJ; ^10^Albert Einstein College of Medicine ‐ Montefiore Medical Center, New York, NY; ^11^NYU Grossman School of Medicine ‐ NYU Brooklyn, New York, NY
3:30 PM	75	**Antimuscarinic Receptor Blockade Reduces Uterine Muscle Contractions Potentially Providing A Novel Treatment For Preterm Labor** **Anthony G. Visco^1^**; Zachary Visco^2^; Chad Grotegut^3^; Cristina Linde^4^; Timothy Westfall^4^; Friederike Jayes^5^ ^1^NinoMed, LLC, Chapel Hill, NC; ^2^University of North Carolina, Chapel Hill, NC; ^3^Wake Forest University, Wake Forest, NC; ^4^Reprocell, Beltsville, MD; ^5^Duke University, Durham, NC
3:45 PM	76	**Vaginal microbiota as a predictor of preterm birth: a prospective cohort study** Laura Lesimple^1^; Jessica Rousseau Rousseau^1^; Luce Landraud^1^; Céline Plainvert^1^; Nathalie Grall^1^; Francois Goffinet^2^; Pierre‐Yves Ancel^1^; Christophe Pannetier^3^; **Laurent Mandelbrot^4^**; Asmaa Tazi^1^ ^1^Assistance Publique Hôpitaux de Paris, Paris, Ile‐de‐France; ^2^Université Paris Cité, Inserm, Centre for Research in Epidemiology and StatisticS (CRESS), Obstetrical Perinatal and Pediatric Epidemiology Research Team (EPOPé), Paris, Ile‐de‐France; ^3^Assistance Publique Hôpitaux de Paris, Paris, Rhone‐Alpes; ^4^Hôpital Louis Mourier, APHP, Université Paris Cité, Colombes, Ile‐de‐France

Saturday, February 1, 2025 • 8:00 AM ‐ 10:15 AM		
Time	Abstract	Oral Concurrent Session 7 ‐ Diabetes
8:00 AM	77	**Does large for gestational age and/or polyhydramnios warrant rescreening for gestational diabetes mellitus?** **Henry Lesser**; A. Dhanya Mackeen; David Chromey, II; Amanda J. Young; Celia Gray; Michael J. Paglia Geisinger Medical Center, Danville, PA
8:15 AM	78	**Breastfeeding patterns among parturients with diabetes: A secondary analysis of the MOMPOD randomized clinical trial** **Minhazur R. Sarker^1^**; Marni B. Jacobs^2^; Kim Boggess^3^; Ashley N. Battarbee^4^; Gladys (Sandy) A. Ramos^1^ ^1^University of California, San Diego, San Diego, CA; ^2^University of California, San Diego Health, San Diego, CA; ^3^University of North Carolina, Chapel Hill, NC; ^4^Center for Women's Reproductive Health, University of Alabama at Birmingham, Birmingham, AL
8:30 AM	79	**Predicting Future Diabetes: Impact of Abnormal Pregnancy OGTT Patterns** **Yael Winter Shafran^1^**; Tal Schiller^2^; Alena Kirzhner^2^; Edi Vaisbuch^2^ ^1^Kaplan Medical Center and REALIFE Research Group, Rehovot, HaMerkaz; ^2^Kaplan Medical Center, Rehovot, HaMerkaz
8:45 AM	80	**Effect of Breastfeeding on the Early Postpartum Lipid Profile in Women with Gestational Diabetes Mellitus** **Harumi Kanzawa^1^**; Hiroshi Yamashita^1^; Ichiro Yasuhi^2^ ^1^NHO Nagasaki Medical Center, Omura City, Nagasaki; ^2^NHO Nagasaki Medical Center, Omura‐City, Nagasaki
9:00 AM	81	**Rates of Risk Appropriate Postpartum Care within Six Months after Delivery** **Jennifer F. Culhane**; Anna Denoble; Olivia Paoletti; Caitlin Partridge; Lisbet S. Lundsberg Yale School of Medicine, New Haven, CT

**Table:** 

Saturday, February 1, 2025 • 8:00 AM ‐ 10:15 AM		
Time	Abstract	Oral Concurrent Session 7 ‐ Diabetes, continued
9:15 AM	82	**Metformin Use in Pregnancy Decreases Neonatal Fat Free Mass** **Claire E. Jensen^1^**; Ashley N. Battarbee^2^; Kim Boggess^1^; Kjersti M. Aagaard^3^ On behalf of the MOMPOD Consortium ^1^University of North Carolina, Chapel Hill, NC; ^2^Center for Women's Reproductive Health, University of Alabama at Birmingham, Birmingham, AL; ^3^Boston Children's Hospital, Division of Fetal Medicine and Surgery, Boston, MA; HCA Healthcare and HCA Healthcare Research Institute, Nashville, TN; HCA Texas Maternal Fetal Medicine, Houston, TX; Baylor College of Medicine and Texas Children's Hospital, Houston, TX, Boston, MA
9:30 AM	83	**Interaction between Metformin and Baseline Insulin Requirements on Neonatal Outcomes in Pregnancies with Type‐2 Diabetes** **Kevin S. Shrestha^1^**; Claire E. Jensen^2^; Kim Boggess^2^; Gladys (Sandy) A. Ramos^3^; Ashley N. Battarbee^4^ ^1^University of Alabama at Birmingham, Birmingham, AL; ^2^University of North Carolina, Chapel Hill, NC; ^3^University of California, San Diego, San Diego, CA; ^4^Center for Women's Reproductive Health, University of Alabama at Birmingham, Birmingham, AL
9:45 AM	84	**The Placental Transcriptome in Pregnancies Complicated by A2 Gestational Diabetes Mellitus** **Morgan E. Wasickanin^1^**; Samantha Carson^1^; Hillary Kinsman^1^; Lydia Bettridge^1^; Katherine E. Free^1^; Jennifer Damicis^1^; Emily Sheikh^1^; Robert Walton^1^; Peter Napolitano^2^; Nicholas Ieronimakis^1^ ^1^Madigan Army Medical Center, Tacoma, WA; ^2^University of Washington, Seattle, WA
10:00 AM	85	**Childhood Sexual and Emotional Maltreatment are Associated with Increased Gestational Weight Gain** **NATALIE E. POLIEKTOV^1^**; Mariana Rocha^1^; Kaitlyn Stanhope^2^; Lauren Holt^1^; Alicia Smith^1^; Vasiliki Michopoulos^1^; Suchitra Chandrasekaran^3^ ^1^Emory University School of Medicine, Atlanta, GA; ^2^Rollins School of Public Health, Atlanta, GA; ^3^Emory University, Atlanta, GA

**Table:** 

Saturday, February 1, 2025 • 10:15 AM – 10:30 AM		
Time	Abstract	Late‐Breaking Abstract Presentations Session 3
10:15 AM	LB05	**Patient navigation to improve postpartum health care among low‐income birthing people: a randomized controlled trial** **Lynn M. Yee^1^**; Joe M. Feinglass^1^; Brittney R. Williams^1^; Laura Diaz^1^; Viridiana Carmona‐Barrera^1^; Ying Cheung^1^; Ka'Derricka Davis^1^; Chen Yeh^1^; Charlotte M. Niznik^1^; Michelle A. Kominiarek^2^; William A. Grobman^3^ ^1^Northwestern University Feinberg School of Medicine, Chicago, IL; ^2^Northwestern Feinberg School of Medicine, Northwestern Feinberg School of Medicine/ Chicago, IL; ^3^Women & Infants Hospital of Rhode Island / Alpert Medical School of Brown University, Brown University/Providence, RI

**Table:** 

Saturday, February 1, 2025 • 8:00 AM ‐ 10:15 AM		
Time	Abstract	Oral Concurrent Session 8 ‐ Fetus and Fetal Intervention
8:00 AM	86	**Innovative transcardiac access to the fetal cerebral and pelvic arteries for diagnosis and treatment** **Michael A. Belfort^1^**; Emma Van Den Eede^2^; Karen Chen^3^; Carolyn A Altman^3^; Timo Krings^4^; Peter T Kan^5^; Tomohiro Arai^2^; Wasinee Tianthong^2^; Walter Coudyzer^6^; Caitlin D. Sutton^3^; Samuel G G. McClugage, III^3^; William E Whitehead^3^; Magdalena Sanz Cortes^1^; Larry H Hollier^3^; Jan A. Deprest^2^; Luc De Catte^6^; Luc Joyeux^3^; Thierry Huisman^3^ ^1^Texas Children's Hospital and Baylor College of Medicine, Houston, TX; ^2^My FetUZ Fetal Research Center, Universitary Hospital Leuven, Leuven, Vlaams‐Brabant; ^3^Baylor College of Medicine and Texas Children's Hospital, Houston, TX; ^4^University Health Network, ON; ^5^UTMB, Houston, TX; ^6^Universitary Hospital Leuven, Leuven, Vlaams‐Brabant

**Table:** 

Saturday, February 1, 2025 • 8:00 AM ‐ 10:15 AM		
Time	Abstract	Oral Concurrent Session 8 ‐ Fetus and Fetal Intervention, continued
8:15 AM	87	**Predicting Outcomes in Congenital Diaphragmatic Hernia with Discordant Fetal MRI and Ultrasound Prognostic Indices** **Lamia A. Alamri^1^**; Jason Gien^1^; Payton Moody^1^; Blair W. Weikel^2^; Mariana Meyers^1^; Michael V. Zaretsky^1^; Sarkis C. Derderian^1^; Henry L. Galan^3^ ^1^University of Colorado, School of Medicine, Aurora, CO; ^2^University of Colorado, School of Medicine and Colorado Fetal Care Center, Aurora, CO; ^3^University of Colorado, School of Medicine, Children's Hospital of Colorado, Aurora, CO
8:30 AM	88	**Tracking twin growth from start to finish in 3D** **Jessica L. Gleason^1^**; Zhen Chen^2^; Rajeshwari Sundaram^2^; Kathryn A. Wagner^3^; Daniel He^4^; Wesley Lee^5^; Roger Newman^6^; Seth Sherman^7^; Edward Chien^8^; Robert Gore‐Langton^7^; Luis F. Goncalves^9^; Katherine L. Grantz^1^ ^1^Epidemiology Branch, NICHD‐NIH, Bethesda, MD; ^2^National Institute of Child Health and Human Development, Bethesda, MD; ^3^Massachusetts College of Pharmacy and Health Sciences, Boston, MA; ^4^The Prospective Group, Fairfax, VA; ^5^Baylor College of Medicine, Houston, TX; ^6^Medical University of South Carolina, Columbia, SC; ^7^The Emmes Company, Rockville, MD; ^8^Cleveland Clinic, Cleveland, OH; ^9^Phoenix Children's Hospital, Phoenix, AZ
8:45 AM	89	**Proteomic analysis of monochorionic pregnancies with twin‐to‐twin transfusion syndrome and selective fetal growth restriction** **Jessian L. Munoz**; Cara Buskmiller; Ahmed A. Nassr; Magdalena Sanz Cortes; Michael A. Belfort; Roopali V. Donepudi Texas Children's Hospital and Baylor College of Medicine, Houston, TX
9:00 AM	90	**Chronotropic rescue prior to cord clamping in fetal complete AV Block and severe bradycardia** **Samantha Holmes^1^**; Emily Bucholz^1^; Camila Londono‐Obregon^1^; Jason Gien^1^; Nicholas Behrendt^1^; Michael V. Zaretsky^1^; Henry L. Galan^2^; Bettina Cuneo^3^ ^1^University of Colorado, School of Medicine, Aurora, CO; ^2^University of Colorado, School of Medicine, Children's Hospital of Colorado, Aurora, CO; ^3^University of Arizona, Tucson, AZ
9:15 AM	91	**Impact of a synthetic amniotic fluid upon fetal lung development** **Stephanie Finoti^1^**; Braxton Forde^2^; Samuel Martin^3^; Marc Oria^1^; Jose L. Peiro^4^ ^1^University of Cincinnati, Cincinnati, OH; ^2^University of Cincinnati College of Medicine, Cincinnati, OH; ^3^Cincinnati Children's Hospital Medical Center, Cincinnati, OH; ^4^Cincinnati Children's Fetal Care Center, Cincinnati, OH
9:30 AM	92	**Effect of very preterm delivery on outcomes following in‐utero spina bifida repair** **Michael V. Zaretsky^1^**; Virginia Lijewski^1^; Jagruti Anadkat^2^; Kelly A. Bennett^3^; Yair J. Blumenfeld^4^; Christopher Q. Buchanan^5^; Caitlin M. Clifford^6^; Timothy Crombleholme^7^; Samer Elbabaa^8^; Stephen P. Emery^9^; William H. Goodnight^10^; Hanmin Lee^11^; Joseph B. Lillegard^12^; Foong‐Yen Lim^13^; Francois Luks^14^; Jena L. Miller^15^; Ueli Moehrlen^16^; Julie Moldenhauer^17^; Anita J. Moon‐Grady^11^; Mauro Schenone^18^; Aimen F. Shaaban^19^; KuoJen Tsao^20^; Tim Van Mieghem^21^; Amy J. Wagner^22^ On behalf of the fMMC Consortium sponsored by NAFTNet ^1^University of Colorado, School of Medicine, Aurora, CO; ^2^Washington University School of Medicine, St. Louis, MO; ^3^Vanderbilt University Medical Center, Nashville, TN; ^4^Children's Hospital at Stanford, Palo Alto, CA; ^5^St. Louis Fetal Care Institute, St. Louis, MO; ^6^University of Michigan Medical Center, Ann Arbor, MI; ^7^Connecticut Children's Fetal Care Center, Hartford, CT; ^8^Orlando Health Arnold Palmer Hospital for Children, Orlando, FL; ^9^University of Pittsburgh, Pittsburgh, PA; ^10^University of North Carolina at Chapel Hill, Chapel Hill, NC; ^11^University of California, San Francisco, San Francisco, CA; ^12^Midwest Fetal Care Center, Minneapolis, MN; ^13^Cincinnati Children's Hospital, Cincinatti, OH; ^14^Fetal Treatment Program of New England, Providence, RI; ^15^Johns Hopkins Medicine, Baltimore, MD; ^16^Children's Hospital Zurich, Zurich; ^17^Children's Hospital of Philadelphia, Philadelphia, PA; ^18^Mayo Clinic, Rochester, MN; ^19^Chicago Institute for Fetal Health, Chicago, IL; ^20^McGovern Medical School at the University of Texas Health Science Center at Houston (UTHealth) University of Texas Health Science Center, Houston, TX; ^21^Mount Sinai Hospital and University of Toronto, Toronto, ON; ^22^Children's Hospital of Wisconsin Fetal Concerns Center, Milwaukee, WI

**Table:** 

Saturday, February 1, 2025 • 8:00 AM ‐ 10:15 AM		
Time	Abstract	Oral Concurrent Session 8 ‐ Fetus and Fetal Intervention, continued
9:45 AM	93	**In‐utero ventriculosubgaleal shunt placement ‐ a fetal lamb model of induced hydrocephalus** Shohra Qaderi^1^; Weston Northam^1^; Soner Duru^2^; Eyal Krispin^3^; Syril James^4^; Jose L. Peiro^5^; Braxton Forde^6^; Hamidreza Forourtan^7^; Ramen H. Chmait^8^; Scott A. Shainker^9^; Cassandra R. Duffy^9^; Nikan Zargarzadeh^1^; Ali Javinani^1^; Arthur Nedder^10^; Brittany Pattison^10^; lana Vasung^10^; Ryne A. Didier^10^; Michaela K. Farber^11^; Sebastian Seifert^12^; Darren B. Orbach^10^; P. Ellen Grant^10^; Benjamin C. Warf^10^; Yves Ville^13^; **Alireza A. Shamshirsaz^14^** ^1^Boston Children's Hospital, Harvard Medical School, Boston, MA; ^2^Cincinnati Children's Hospital, Cincinnati, OH; ^3^Harvard Medical School, Boston, MA; ^4^Hôpital Necker‐Enfants Malades, Paris, Ile‐de‐France; ^5^Cincinnati Children's Fetal Care Center, Cincinnati, OH; ^6^University of Cincinnati College of Medicine, Cincinnati, OH; ^7^Laparoscopy research center, Shiraz, Fars; ^8^Keck School of Medicine, University of Southern California, Los Angeles, CA; ^9^Beth Israel Deaconess Medical Center, Boston, MA; ^10^BCH, Boston, MA; ^11^BWH, Boston, MA; ^12^Brigham & Women's Hospital, Boston, MA; ^13^University and Necker‐Enfants Malades Hospital, Paris, Ile‐de‐France; ^14^Boston Children's Hospital, Brigham and Women's Hospital, Beth Israel Deaconess Medical Center, Harvard Medical School, Boston, MA
10:00 AM	94	**Role of placental superficial anastomoses in twin‐twin transfusion syndrome (TTTS)** **Ramen H. Chmait^1^**; Lisa M. Korst^2^; Arlyn Llanes^1^; Kristine R. Rallo^1^; Andrew H. Chon^3^; Martha A. Monson^4^; Ruben A. Quintero^5^ ^1^Keck School of Medicine, University of Southern California, Los Angeles, CA; ^2^Childbirth Research Associates, North Hollywood, CA; ^3^Oregon Health & Science University, Portland, OR; ^4^Intermountain Healthcare, University of Utah Health, Salt Lake City, UT; ^5^The Fetal Institute, USFETUS Research Consortium, Miami, FL

**Table:** 

Saturday, February 1, 2025 • 10:15 AM – 10:30 AM		
Time	Abstract	Late‐Breaking Abstract Presentations Session 4
10:15 AM	LB06	**Comparing 162mg vs. 81mg Aspirin for Prevention of Preeclampsia (ASAPP): A Randomized Control Trial** **Amrin Khander^1^**; Kathy Matthews^2^; Charlene Thomas^3^; Paul Christos^4^; Stephen T. Chasen^5^; Daniel W. Skupski^3^; Laura E. Riley^3^; Phyllis August^6^; Line Malha^3^ ^1^Weill Cornell Medicine‐New York Presbyterian Hospital, New York, NY, NY; ^2^New Jersey Perinatal Associates, Livingston, NJ; ^3^Weill Cornell Medicine, New York, NY; ^4^Weill Cornell Medical College, New York, NY; ^5^Weill‐Cornell Medical College, New York, NY; ^6^New York Presbyterian Weill Cornell Medicine, New York, NY

**Table:** 

Saturday, February 1, 2025 • 8:00 AM ‐ 10:30 AM		
Time	Abstract	Oral Concurrent Session 9 ‐ Medical Complications
8:00 AM	95	**Prevention Of Methamphetamine Use among Postpartum People (PROMPT): A Pilot Randomized Controlled Trial** **Marcela C. Smid^1^**; Jasmin E. Charles^1^; Amanda A. Allshouse^2^; Stephanie Castro^1^; Grace Humiston^1^; Elysha Cash^2^; Adam G. Gordon^2^; Kristi Carlston^1^; Marie Gibson^1^; Gerald Cochran^1^ ^1^University of Utah Health, Salt Lake City, UT; ^2^University of Utah, Salt Lake City, UT
8:15 AM	96	**Postpartum VTE risk by CHA2DS2‐VASc score in patients with atrial fibrillation or flutter during pregnancy** **Virginia Y. Watkins^1^**; Miriam L. Estin^2^; Sarah C. Snow^1^; Cary C. Ward^1^; Marie‐Louise Meng^1^; Jerome J. Federspiel^1^ ^1^Duke University School of Medicine, Durham, NC; ^2^Duke university School of Medicine, Durham, NC

**Table:** 

Saturday, February 1, 2025 • 8:00 AM ‐ 10:30 AM		
Time	Abstract	Oral Concurrent Session 9 ‐ Medical Complications, continued
8:30 AM	97	**A Technology Enabled Solution for Perinatal Mental Health Collaborative Care Models: A Randomized Controlled Trial** **Emily S. Miller^1^**; David Mohr^2^; Dinah Williams^3^; Melissa Shikany^2^; Tracy Walsh^2^; Nathan W. Winquist^2^; Zara Mir^2^; Elizabeth L. Gray^2^; Shannon R. Smith^2^; Charles Krause^2^; Lara M. Baez^2^; Madhu C. Reddy^4^ ^1^Women & Infants Hospital of Rhode Island and Alpert Medical School of Brown University, Providence, RI; ^2^Northwestern University Feinberg School of Medicine, Chicago, IL; ^3^Women and Infants Hospital of Rhode Island, Providence, RI; ^4^University of California, Irvine, Irvine, CA
8:45 AM	98	**Risk of severe maternal morbidity among pregnant people with colorectal cancer using a population database** **Shriddha Nayak^1^**; Kristin C. Darwin^2^; Arthur J. Vaught^2^; Marika Toscano^1^ ^1^Johns Hopkins University, School of Medicine, Baltimore, MD; ^2^Johns Hopkins University, Baltimore, MD
9:00 AM	99	**Impact of breastfeeding on estimated risk of postpartum cardiovascular disease** **Christine P. Field^1^**; William A. Grobman^1^; Jiqiang Wu^1^; Anna Palatnik^2^; Mark B. Landon^1^; Denise Scholtens^3^; William Lowe^3^; Nilay S. Shah^4^; Jami Josefson^3^; Sadiya S. Khan^5^; Kartik K. Venkatesh^1^ ^1^The Ohio State University, Columbus, OH; ^2^Medical College of Wisconsin, Milwaukee, WI; ^3^Northwestern University, Northwestern University/Chicago, IL; ^4^Northwestern University, Chicago, IL; ^5^Northwestern University Feinberg School of Medicine, Chicago, IL
9:15 AM	100	**Intravenous Ferumoxytol versus Oral Ferrous Sulfate for Iron‐Deficiency Anemia in Pregnancy: A Randomized Controlled Trial** **Irogue I. Igbinosa**; Stephanie A. Leonard; Ijeoma Iweakogwu; Elizabeth B. Sherwin; Caroline Berube; Deirdre J. Lyell Stanford University, Palo Alto, CA
9:30 AM	101	**Neurodevelopmental outcomes of 3‐year‐old children of mothers with SARS‐CoV‐2 infection in pregnancy** **Lydia L. Shook**; Victor Castro; Laura Ibanez‐Pintor; Roy H. Perlis; Andrea G. Edlow Massachusetts General Hospital, Boston, MA
9:45 AM	102	**Maternal Gestational Weight Gain in GLP‐1 Agonist Exposed and Unexposed Patients** Nishita Pondugula^1^; Jennifer F. Culhane^1^; Lisbet S. Lundsberg^1^; Caitlin Partridge^1^; **Audrey A. Merriam^2^** ^1^Yale School of Medicine, New Haven, CT; ^2^Yale New Haven Hospital, New Haven, CT
10:00 AM	103	**Placental Pathology Findings by Timing and Amount of Maternal Cannabis Exposure** **Shilpa S. Tummala^1^**; Amanda A. Allshouse^2^; Gwendolyn A. McMillin^1^; Jessica M. Comstock^2^; Elizabeth S. Doughty^2^; Robert M. Silver^2^; Torri D. Metz^2^ ^1^University of Utah Health, Salt Lake City, UT; ^2^University of Utah, Salt Lake City, UT
10:15 AM	104	**The Impact of a Facilitated Postpartum‐to‐Primary Care Transition on Care Utilization within One Year Postpartum** **Arlin Delgado^1^**; Pichliya Liang^1^; Tierra Bender^1^; Kaitlyn E. James^1^; Alaka Ray^1^; Ishani Ganguli^2^; Jessica L. Cohen^3^; Mark A. Clapp^1^ ^1^Massachusetts General Hospital, Boston, MA; ^2^Brigham and Women's Hospital, Boston, MA; ^3^Harvard T.H. Chan School of Public Health, Boston, MA

